# Classification of schizophrenia patients based on resting-state functional network connectivity

**DOI:** 10.3389/fnins.2013.00133

**Published:** 2013-07-30

**Authors:** Mohammad R. Arbabshirani, Kent A. Kiehl, Godfrey D. Pearlson, Vince D. Calhoun

**Affiliations:** ^1^The Mind Research NetworkAlbuquerque, NM, USA; ^2^Department of ECE, University of New MexicoAlbuquerque, NM, USA; ^3^Department of Psychology and Neuroscience, University of New MexicoAlbuquerque, NM, USA; ^4^Olin Neuropsychiatry Research CenterHartford, CT, USA; ^5^Department of Psychiatry, Yale University School of MedicineNew Haven, CT, USA

**Keywords:** functional network connectivity, independent component analysis (ICA), classification, schizophrenia, resting-state fMRI

## Abstract

There is a growing interest in automatic classification of mental disorders based on neuroimaging data. Small training data sets (subjects) and very large amount of high dimensional data make it a challenging task to design robust and accurate classifiers for heterogeneous disorders such as schizophrenia. Most previous studies considered structural MRI, diffusion tensor imaging and task-based fMRI for this purpose. However, resting-state data has been rarely used in discrimination of schizophrenia patients from healthy controls. Resting data are of great interest, since they are relatively easy to collect, and not confounded by behavioral performance on a task. Several linear and non-linear classification methods were trained using a training dataset and evaluate with a separate testing dataset. Results show that classification with high accuracy is achievable using simple non-linear discriminative methods such as k-nearest neighbors (KNNs) which is very promising. We compare and report detailed results of each classifier as well as statistical analysis and evaluation of each single feature. To our knowledge our effects represent the first use of resting-state functional network connectivity (FNC) features to classify schizophrenia.

## Introduction

Population studies show that lifetime prevalence of all psychotic disorders is as high as 4% (http://www.nimh.nih.gov/statistics/SMI_AASR.shtml). These disorders can impair normal life significantly and impose huge societal cost (Rice, 1999). Clinically, the patient's self-reported experiences and observed behavior over the longitudinal course of the illness constitute the basis for diagnosis. The overlapping symptoms of mental disorders and the absence of standard biologically-based clinical tests make differential diagnosis a challenging task. Early diagnosis of these diseases can significantly improve treatment response and reduce associated costs (McGlashan, 1998).

Advances in neuroimaging technologies in the past two decades have opened a new window into the structure and function of the healthy human brain as well as illuminating many brain disorders such as schizophrenia. Schizophrenia is among the most prevalent mental disorders affecting about 1% of the population worldwide (Wyatt et al., 1995; Bhugra, 2005). This devastating, chronic heterogeneous disease is usually characterized by disintegration in perception of reality, cognitive problems and chronic course with lasting impairment (Heinrichs and Zakzanis, 1998). Multiple structural and functional brain abnormalities are widely reported in patients with schizophrenia (Shenton et al., 2001; Calhoun et al., 2009a; Karlsgodt et al., 2010). Most neuroimaging-based studies of schizophrenia focus on showing aberrations of some features (structural or functional) in a patient group by comparing them to a control group. While many of these findings are statistically significant in the average sense, discrimination ability of those features is under question for classification purposes on a case-by-case basis. Since classification provides information for each individual subject, it is considered a much harder task than reporting group differences. In the case of classifying schizophrenia patients, a small number of training samples (subjects) and high dimensional data make it a challenging task to design an accurate, robust classifier for such a heterogeneous brain disorder.

Recently, there is a growing interest in designing objective prognostic/diagnostic tools based on neuroimaging and other data that display high accuracy and robustness. The relatively small amount of research on MRI-based classification of schizophrenia patients can be divided into three categories based on the type of discriminating features used: structural-based (Csernansky et al., 2004; Nakamura et al., 2004; Davatzikos et al., 2005; Fan et al., 2005, 2007b; Caan et al., 2006; Pardo et al., 2006; Kawasaki et al., 2007; Yoon et al., 2007; Caprihan et al., 2008; Sun et al., 2009; Takayanagi et al., 2010, 2011; Ardekani et al., 2011), functional-based (Georgopoulos et al., 2007; Calhoun et al., 2008b; Demirci et al., 2008a; Michael et al., 2008; Arribas et al., 2010; Shen et al., 2010; Castro et al., 2011) or combination of structural and functional features (Fan et al., 2007a; Ford et al., 2002).

In recent years, spontaneous modulation of blood oxygenation level-dependent (BOLD) signal during the resting condition has found fruitful clinical applications (Fox and Greicius, 2010). Resting-state fMRI (rfMRI) experiments are less prone to multi-site variability, allow a wider range of patients to be scanned and make it possible to study multiple cortical systems from one dataset (Fox and Greicius, 2010). Moreover, more accurate connectivity maps can be detected using rfMRI data compared to task-based fMRI data (Xiong et al., 1999). With considerable literature on rfMRI group comparisons, researchers have started tackling more challenging task of using the found abnormalities or so called biomarkers to discriminate patients from healthy controls. The main target of these studies has been the Alzheimer's disease (Li et al., 2002; Greicius et al., 2004; Wang et al., 2006; Supekar et al., 2008). However, rfMRI data have been rarely used for discrimination of schizophrenia (Cecchi et al., 2009; Shen et al., 2010; Du et al., 2012). Shen et al. (2010) used an atlas-based method to extract mean time-courses of 116 brain regions in the resting-state for both healthy controls and schizophrenia subjects. The correlation between these time-courses made the feature vector for each subject. By using feature selection and dimensionality reduction techniques, they reduced the dimensionality down to three where they classified patients from controls with a high accuracy (93% for patients and 75% for healthy controls).

The main purpose of this study is using resting-state functional network connectivity (FNC) features for classification of schizophrenia patients. Using functional connectivity (FC) methods, researchers have shown disrupted functional integration in schizophrenia patients (Friston and Frith, 1995; Frith et al., 1995; Josin and Liddle, 2001; Bokde et al., 2006; Mikula and Niebur, 2006; Salvador et al., 2010). Liang et al. reported decreased FC among insula, prefrontal lobe and temporal lobe and increase connectivity between cerebellum and several other brain regions. Meyer-Lindenberg et al. (2001) reported abnormal FC in fronto-temporal interactions in schizophrenia in selected regions of interest (ROIs) using positron emission tomography (PET) brain scans on working memory task. Salvador et al. (2010) reported hyper-connectivity within medial and orbital structures of the frontal lobe and hyper-connectivity between these regions and several cortical and sub-cortical structures in schizophrenia patients. FC is defined as correlation (or other kinds of statistical dependency) among spatially remote brain regions (Friston, 2002). FC analysis documents interactions among brain regions during a task as well as during rest. Two widely used FC approaches are: (a) seed-based analysis (Biswal et al., 1995, 1997; Lowe et al., 1998; Cordes et al., 2000, 2002; Stein et al., 2000; Ford et al., 2005) and (b) spatial independent component analysis (ICA) (McKeown et al., 1998; Calhoun et al., 2001a; van de Ven et al., 2004; Esposito et al., 2005; Garrity et al., 2007). In the seed-based approach, individual seed voxels from predefined brain regions of interest (ROI) are chosen and the cross correlation of other voxels' time courses (TCs) with the selected seeds then computed, to derive a correlation map. This map can then be thresholded to identify voxels showing significant FC with the seed voxels.

An alternative approach is based on ICA, a multivariate data-driven method which as a blind source separation method, can recover a set of signals from their linear mixtures and has yielded fruitful results with fMRI data (Calhoun et al., 2009b; Calhoun and Adali, 2012). ICA estimates maximally independent components using independence measures based on higher-order statistics. Compared to general linear model approaches, ICA requires no specific temporal model (task-based design matrix), making it ideal for analyzing resting state data (Kiviniemi et al., 2003). Depending on data matrix formation, one can perform either temporal or spatial ICA (sICA) on fMRI data. sICA is the predominant ICA approach used for fMRI data (McKeown et al., 1998; Calhoun et al., 2001a,b). sICA decomposes fMRI data into a set of maximally spatially independent maps and their corresponding time-courses. Each thresholded sICA map may consist of several remote brain regions forming a brain functional network. sICA generates consistent spatial maps (SMs) while modeling complex fMRI data collected during a task or in the resting-state (Turner and Twieg, 2005) although the task can result in a subtle modulation of the spatial patterns (Calhoun et al., 2008a). The dynamics of the BOLD signal within a single component is described by that component's TC. Regions contributing significantly within a given component are strongly functionally connected to each other.

There is growing interest in studying FC among brain functional networks. This type of connectivity, which can be considered as a higher level of FC, is termed FNC (Jafri et al., 2008) and measures the statistical dependencies among brain functional networks. Each functional network may consist of multiple remote brain regions. Spatial components resulting from sICA are maximally spatially independent but their corresponding time-courses can show a considerable amount of temporal dependency. This property of sICA makes it an excellent choice for studying FNC, which can be studied by analyzing these weaker dependencies among sICA TCs. These dependencies can be analyzed by correlation methods (Jafri et al., 2008) or algorithms such as dynamic causal modeling (Stevens et al., 2007) or Granger causality (Stevens et al., 2009; Havlicek et al., 2010).

It has been shown that there are significant FNC differences between schizophrenic patients and the control group in the resting-state possibly showing deficiencies in the brain functional processing in the patients (Jafri et al., 2008; Calhoun et al., 2009a, 2011). Jafri et al. (2008) reported increased FNC among frontal, temporal, visual and default-mode networks and decreased FNC between temporal and parietal networks. We hypothesized that disrupted functional integration in schizophrenia patients as captured by FNC analysis entail valuable information that can be used to discriminate patients automatically. To test our hypothesis we conducted a feasibility study of using FNC features for classification of schizophrenia patients to our knowledge for the first time. In order to show that our method can provide significant results regardless of the type of machine learning algorithm, we report the results for several linear and non-linear classification methods such as minimum least square linear classifier, Fisher's linear discriminant classifier (LDC), quadratic classifier, binary decision tree, support vector machine (SVM), k-nearest neighbor (KNN), artificial neural networks (ANN), naïve Bayes, logistic linear classifier (LLC) and dissimilarity-based classifier. Careful considerations were taken to avoid common pitfalls in automatic classification studies such as using very small cohort, using testing dataset information in the training phase and incomplete report of the results (Demirci et al., 2008b). The results show that the proposed method can classify the schizophrenia patients with very high specificity and sensitivity.

## Materials and methods

### Participants and paradigm description

One session of resting-state fMRI data was collected from 28 healthy and 28 schizophrenic patients. Participants gave written, informed, Hartford hospital and Yale IRB approved consent at the Institute of Living and were compensated for their participation. Schizophrenia was diagnosed according to the DSM-IV TR criteria on the basis of a structured clinical interview (SCID) (First et al., 1995) administered by a research nurse and review of the medical file. Exclusion criteria included any participants with auditory or visual impairment, mental retardation (full scale IQ < 70), traumatic brain injury with loss of consciousness greater than 15 min, presence or history of any central neurological illness and a positive urine pregnancy test. Participants were also excluded if they met criteria for alcohol or drug dependence within the past 6 months or produced a positive (assessed by urine toxicology screen on the day of scanning). Although patients were slightly older than controls (SZ age = 39.7 ± 10.1; HC age = 36.5 ± 11.3), the difference was not statistically significant (two sample *t*-test *p*-value: 0.27). All but three patients and one control were right handed. Healthy participants were free of any DSM-IV TR Axis I disorder (SCID) or psychotropic medication and had no first-degree relatives with a psychotic illness.

### Image acquisition

Scans were acquired at the Olin Neuropsychiatry Research Center at the Institute of Living/Hartford Hospital on a Siemens Allegra 3T dedicated head scanner equipped with 40 mT/m gradients and a standard quadrature head coil. The transaxial functional scans were acquired using gradient-echo echo-planar-imaging with the following parameters (repeat time (TR) = 1.50 s, echo time (TE) = 27 ms, field of view = 24 cm, acquisition matrix = 64 × 64, flip angle = 70°, voxel size = 3.75 × 3.75 × 4 mm^3^, slice thickness = 4 mm, gap = 1 mm, 29 slices, ascending acquisition). Six “dummy” scans were performed at the beginning to allow for longitudinal equilibrium, after which the paradigm was automatically triggered to start by the scanner. The resting state scan consisted of one 5 min run.

### Proposed approach

**Figure 1:**
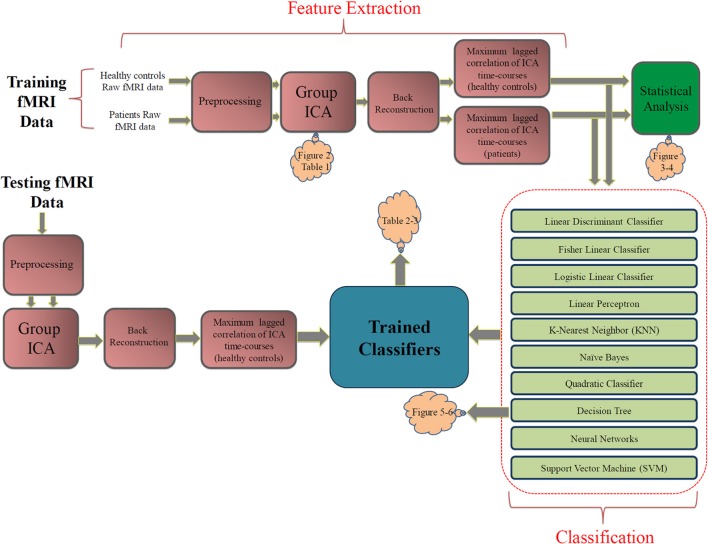
**The proposed approach**. The pink blocks on the top show the feature extraction steps. The statistical analysis box (green) is not part of the classification approach. The light green blocks describe the classification stage. Orange clouds indicate the corresponding figures and tables in the Results section.

The block diagram in Figure 1 shows our approach. We divided the data into separate training (16 healthy subjects + 16 patients) and testing (12 healthy subjects + 12 patients) randomly. The raw fMRI data was first preprocessed. Then the training data were analyzed with group ICA. Subject specific SMs and time-courses were computed using back reconstruction. Next, FNC analysis was performed on the subject specific ICA time-courses. FNC was calculated between each pair of selected components.

Several classifiers were trained using the training data and were evaluate using the testing data. Leave-one-out cross validation (LOOCV) inside the training set was used to select the hyperparameters for the classifiers. The optimum parameters for relevant classifiers were selected based on the averaged validation error over 32 validation iterations. In the testing phase, a separate ICA was performed on the testing dataset and the extracted brain networks where matched with those of training ICA based on maximum Pearson correlation coefficient. Finally, performances of trained classifiers were evaluated using the testing features.

As a supplementary study, the FNC features were statistically analyzed within each group of subjects using one sample *t*-tests and between groups using two-sample *t*-tests on the training dataset. Statistical test within each group test the null hypothesis that each feature has a mean of zero. Features surviving the test have non-zero mean which is statistically significant (which tells us there is a significant correlation between the pair of components). Two sample *t*-tests between groups test the null hypothesis that corresponding FNC features in the two groups (controls and patients) have the same mean. Features surviving this test are the ones significantly (from a statistical point of view) different between control and patient groups (and tell us that the correlation between the pair of components is greater in one group compared to the other group). Note that these results are presented for descriptive purposes but were not used for feature selection or at all in the classification process. Each of the blocks in Figure 1 is described in more details in the following subsections.

#### Preprocessing

Data were preprocessed using SPM5 software (http://fil.ion.ucl.ac.uk), motion corrected, spatially normalized into standard MNI space and slightly subsampled to voxel size 3 × 3 × 3 mm^3^, resulting in 53 × 63 × 46 voxels. Next, spatial smoothing with a 10 × 10 × 10 mm^3^ FWHM Gaussian kernel was performed.

#### Group ICA and back reconstruction

Prior to the ICA, data dimensionality was reduced at two levels using principal component analysis (PCA). First at the subject level, dimensionality was reduced to 80. Then reduced data from all subjects and all sessions were concatenated together and put through another reduction step. The number of components for the second level reduction was estimated to be 20 by minimum description length (MDL) criterion (Li et al., 2007). This is also the number of IC components. Note the MDL is a data driven approach, so it is not dependent on whether data are collected at rest or during a task.

Infomax group sICA (Calhoun et al., 2001a) was conducted to decompose the aggregated data into components using GIFT software (http://icatb.sourceforge.net/). SICA applied to fMRI data identifies temporally-coherent networks (TCNs) by estimating maximally independent spatial sources, referred to as SMs and their corresponding TCs.

In order to validate the number of ICA components chosen by MDL and also measure the robustness of each of them, ICA was repeated 10 times using ICASSO (http://www.cis.hut.fi/projects/ica/icasso). Each time, the ICA algorithm was started from a different initial point and the resulting components were clustered to estimate the reliability of the decomposition (Himberg et al., 2004). Robustness and reliability of components were well validated by ICASSO results showing compact clusters.

In order to estimate subject-specific SMs and TCs, a back-reconstruction approach based on PCA compression and projection was used (Calhoun et al., 2001b; Erhardt et al., 2010). Subject-specific TCs were reconstructed separately for patients and controls.

#### Component selection

SMs were reconstructed and converted to *Z* values for each of the subjects. All of the components were visually inspected and the non-artifactual components were selected. Non-artifactual components are expected to have peak activation in the gray matter and have low spatial overlap with known ventricles, vascular, motion and susceptibility artifacts.

#### Functional network connectivity

The FNC toolbox (http://mialab.mrn.org/software/#fnc) was used for the FNC analysis. As mentioned before, significant temporal correlation can exist among the sICA TCs. The FNC toolbox computes maximum lagged correlation among the components. The maximum lagged correlation was computed as in (Jafri et al., 2008). First the TCs of the ICA components were interpolated to allow us detection of any delays less than the TR of the scanner (Calhoun et al., 2000; Ford et al., 2005). We assume ρ for the Pearson correlation coefficient between two TCs named $\bar{X}$ and $\bar{Y}$ of dimension *T* × 1 where *T* is the number of time points in TCs. Starting reference point of the TCs is named *i*_*o*_ and Δ*i* represents the non-integer change in time. ρ_Δ*i*_ represents the Pearson correlation between ${\bar{X}}_{i_{0}}$ which is vector at the reference time point *i*_*o*_ and ${\bar{Y}}_{i_{0}+\Delta i}$ which is vector $\bar{Y}$ shifted Δ*i* from the reference time point. This correlation between the overlapping points of ${\bar{X}}_{i_{0}}$ and ${\bar{Y}}_{i_{0}+\Delta i}$ can be computed as follows:

(1) $$\rho_{\Delta i}=\frac{\left({\bar{X}}_{i_{0}}^{T}\right)({\bar{Y}}_{i_{0}+\Delta i})}{\sqrt{{\bar{X}}_{i_{0}}^{T}{\bar{X}}_{i_{0}}}\times \sqrt{{\bar{Y}}_{i_{0}\;+\Delta i}^{T}{\bar{Y}}_{i_{0}+\Delta i}}}$$

The ρ_Δ*i*_ vector is calculated for each pair of TCs when one of TCs is shifted Δ*i* units from −3 to +3 s (i.e., ± 2 TR). The maximum correlation and the corresponding lag is calculated and saved for each of the subjects and separately for rest and task. Allowing lag between signals is important to account for variations in hemodynamic response shapes among brain regions as well as among subjects. Although the lag can give an idea of temporal order of fMRI TCs, the source of the lag is not completely understood and could be due to mixture of functional and physiological effects. For these reasons, we will not report any analysis on the lag parameter in this paper. The lag corresponding to the maximum correlation was checked to be distributed in ±3 s interval and often away from its maximum or minimum.

Prior to computing correlations, ICA TCs were filtered. There are reports that show task related and other interesting information resides in lower frequencies while noise and artifacts contributes mostly to the higher frequency contents of the TCs (Cordes et al., 2001). We applied a bandpass Butterworth filter with cut-off frequencies at 0.017 Hz and 0.15 Hz to the ICA TCs. Also, we regressed out the motion parameters from the FNC values to remove any movement bias from the analysis.

#### Statistical analysis

For all FNC analyses, correlations were transformed to *z*-scores using Fisher's transformation [*z* = arctanh(*r*)]. Then, robustness of maximum lagged correlation between each pair of TCs was tested separately for rest and task using *t*-tests. Finally, to determine the significant differences of rest versus task, paired *t*-tests were conducted on the two groups. The cut-off *p*-value for all of the tests was set at *p* < 0.05 and was corrected for multiple comparisons using the false discovery rate (FDR) method (Genovese et al., 2002).

#### Classification

We evaluated the performance of several well-known linear and non-linear classifiers. This will give us a better view of the complexity of the features. If simpler classifiers (such as linear classifiers) classify the data successfully, it means that the features have a simple structure (classes are almost linearly separable). However, if just complicated non-linear classifiers classify the data successfully, it is an indication that data has a more complex structure. The decision boundary in a linear classifier is a hyperplane while in a non-linear classifier the boundary can take any shape. In another sense, the classifiers can be divided into generative and discriminative. In generative classifiers, the probability density functions (pdf) of all classes are modeled and the Bayes theorem gives the posterior probabilities. On the other hand, discriminative classifiers try to estimate the posterior probability directly or skip the challenging step of pdf estimation and determine the decision boundary based on the observed data (discriminant methods). Generative methods are often simpler and more computationally efficient but require estimation of pdf which require substantial amount of data. For complex data sets with few training samples, discriminative methods yield a better performance. It should be noted that in this study we computed the prior probabilities for the two classes from the data (which is equal) since the distribution of the data is very different from the real prevalence of schizophrenia (around 1%). All classifiers were implemented using Matlab (MathWorks, Inc.). Naïve Bayes, logistic linear and quadratic classifiers along with decision trees (DT) were implemented using PRTools (http://www.prtools.org) which is a Matlab-based pattern recognition toolbox (Duin et al., 2007). In this section, these methods will be briefly reviewed.

***Linear methods.***

*Linear Bayes normal classifier*. This simple classifier assumes Gaussian pdf for both classes with equal covariance matrices but different means. The joint covariance matrix is the weighted average of class covariance matrices (weighted by prior probabilities). Using the Bayes rule, these assumptions lead to a linear decision boundary. This classifier is also called LDC (Duda et al., 2001).

*Fisher linear classifier (FLC)*. Fisher's linear discriminant views classification as a dimensionality reduction task. Fisher formulation tries to maximize class mean separation while minimizing class overlap during linear dimension reduction. This choice of direction for projection can be used as a linear classifier in a two class problem. Fisher' linear classifier is special case of minimum least square linear classifier (Bishop, 2006).

*Logistic linear classifier (LLC)*. Logistic regression in method of learning functions from *f* : *X* → *Y*. *X* = [*X*_1_ *X*_2_ … *X*_*n*_] is the training vector with *n* variables and is the target value (class). Logistic regression assumes a parametric for the distribution *P*(*Y*|*X*). The parameters are estimated from the training data. Assuming that is binary (two class problem), the logistic regression can be formulated as below:

(2) $$P(Y=0|X=\frac{\text{exp}\left(w_{0}+\sum_{i=1}^{n}w_{i}X_{i}\right)}{1+\text{exp}\left(w_{0}+\sum_{i=1}^{n}w_{i}X_{i}\right)}$$

(3) $$P(Y=1|X)=\frac{1}{1+\exp(w_{0}+\sum_{i=1}^{n}w_{i}X_{i})}$$

One of the nice properties of the logistic regression is its ability to provide a linear discriminant between the two classes. Each new object is assigned to a class that has a larger probability for that object. Simplifying this rule results in a classification rule:

(4) $$if\;w_{0}+\sum_{i=1}^{n}w_{i}X_{i}>0\to Y=0\text{ Otherwise }Y=1$$

LLC also provides the weight for each feature so it can be used to rank the features.

*Linear perceptron classifier*. This classic linear discriminant tries to minimize the error function which is the number of misclassifications. This classifier can be considered as simple feed forward ANN (Rosenblatt, 1958). First the input vector is transformed using a non-linear transformation to give a feature vector. The algorithm then tries to change the weight vector of the neural network using gradient stochastic descent algorithm to minimize the error in an iterative manner. At each iteration, the weight vector of the network is manipulated by perceptron learning rule. The perceptron convergence theorem guarantees that the perceptron learning algorithm can find the solution in finite number of steps if such a solution (data is linearly separable) exists (Block et al., 1962).

*Linear support vector machine (SVM)*. Over the last 15 years following the work by Cortes et al. (Cortes and Vapnik, 1995), SVM has proven useful in many machine learning and pattern recognition analysis problems. Moreover, when data classes are heterogeneous with few training samples, SVMs appear to be especially beneficial (Melgani and Bruzzone, 2004). This binary classifier aims at finding a hyperplane that maximizes the margin between the two classes. The training samples closest to the decision boundary are called support vectors. By allowing a margin (called soft margin) that allows for misclassification of some noisy samples, SVMs avoid the overfitting problem.

***Non-linear methods.***

*K-nearest neighbor*. KNN is a method of classifying objects based on proximity to the training samples (Cover and Hart, 1967). This instance-based learning method is among the simplest machine learning approaches. Each object is classified by the majority voting of the training samples in the neighborhood. The most common class among the *k* nearest neighbors is determined and is assigned to the object (Bremner et al., 2005). KNN can result in complex decision boundaries. The optimum *k* is determined by cross validation. Different distance metrics such as Euclidean, city block, cosine and correlation can be used to measure the proximity of the samples. KNN is fast, simple and guarantees an error rate no worse than twice the Bayes error if the amount of data approach infinity. We used just Euclidean distance metric in our analysis.

*Naïve Bayes classifier (NBC)*. The naïve Bayes classifier is a simple generative classifier based on Bayes theorem. The naïve assumption of NBC is that it assumes independence among the features. Although this over-simplified assumption is violated in most of the machine learning problems, this approach worked very well for many complex problems even when the independence assumption is not valid (Domingos and Pazzani, 1997; Rish, 2001). One of the main advantages of NBC is that it requires small amount of data to estimate the parameters of pdf function for each feature. Since the features are assumed to be independent, the joint pdf of the features is simply the multiplication of individual pdfs of each feature. When dealing with continuous data, typically Gaussian distribution is assumed for each feature. The pdf parameters are estimated from the training data. NBC works quite well in anti-spam filtering problems (Seewald, 2007).

*Quadratic Bayes normal classifier*. Quadratic discriminant analysis (QDC) is closely related to linear discriminant analysis. It assumes that the data is normally distributed with different mean and covariance matrices. This results in a quadratic decision boundary (Duda et al., 2001).

*Binary decision tree*. DT find use in a wide range of applications. DT partitions the input space into cubic regions. In classification a class label is assigned to each region in the input space. Interpretability of the DT makes them very popular specially in medical diagnosis (Bishop, 2006). Each decision is a result of a sequence of binary decisions. In order to learn a model from the training samples, the structure of the tree and the threshold value for each node should be determined. There are many variations of DT but most of them rely on the top–down greedy search in the space of possible trees called ID3 algorithm (Quinlan, 1987) and its successor C4.5 (Quinlan, 1993). Selecting optimal tree structure is usually infeasible due to large number possibilities. Usually the tree is started with a single root node and then at each step one node is added to the tree. This is called greedy strategy for growing the tree. At each node an attribute (feature) should be selected to be tested. There are several criteria to measure the worth of each feature such as information gain, diversity index, Fisher's criterion (the same used in Fisher discriminant analysis) and gain ratio. The threshold values and structure of the tree is chosen so that the classification error is minimized. A criterion to stop growing the tree (pruning) should also be devised. Often the tree is fully grown and then the tree is pruned back to find the best tree for that structure. Graphical representation and human interpretability of the DT makes them very popular. However, since the edges of the decision regions are aligned with the axis of the feature space they are very suboptimal (Bishop, 2006). One of the main advantages of DT is interpretability. Moreover, they show the importance of each feature for classification in a graphical illustration.

*Artificial neural networks*. Multilayer ANN is the extension of linear perceptron classifier. These networks can result in complex non-linear decision boundaries. A well-known structure for a tree layer structure: Input layer, hidden layer and output layer. Each neuron in each layer has connections to other neurons of the subsequent layers. Non-linear transfer function of the neurons in the hidden layer can take any form such as sigmoid. The weights of the nodes are changed using a technique called backpropagation (Werbos, 1990). At each iteration, the output of the network is compared to correct answers and based on a predefined error function, an error value is computed. This error is fed back to the network and the weights of each node are adjusted to minimize this error. This can be done by gradient descent technique if the activation function is differentiable. Other method of minimizing the error is using Levenberg–Marquardt algorithm (Levenberg, 1944).

Another class of ANN uses radial basis activation function in the hidden layer (Chen et al., 1991). Usually this kind of network requires more neurons than standard feed forward back-propagation network but can be trained much faster. Topology of ANN used in this study can be found in the Results section.

*Non-linear support vector machine*. By using the kernel trick, SVM can map the not-linearly separable data into a higher dimensional space where the samples are hopefully lineally separable. This mapping to higher dimensional space is difficult, but since SVM formulation depends on the inner product of each of training samples with the support vectors, the kernel is defined as this inner product so the problem is solved in the same fashion as the linear case. There are many kernel functions but the most widely used ones are Gaussian radial basis function (RBF) and polynomial kernel. There is at least one parameter in a kernel (except for the linear kernel) which should be optimized along with the soft margin usually by grid search over reasonable values of that parameter. RBF and polynomial kernels are defined as below:

(5) $$K(X_{i},X)=\text{exp}\left(-\frac{\left\|x_{i}-x\right\|}{\sigma }\right)$$

(6) $$K(X_{i},X)={\left[x_{i}.x+1\right]}^{p}$$

In the above equations, support vectors are denoted by *x*_*i*_ and each training point is denoted by *x*. σ is a parameter proportional to the width of the RBF kernel. *p* is the degree of the polynomial kernel. A detailed mathematical formulation of SVM can be found in Burges (1998).

#### Parameter selection

The parameters for each classifier were selected by grid search. Unfortunately, there is no exact theoretical solution for the optimum value for most of the parameter. The parameters were selected based on the average validation error.

### Effect of medication

One limitation of this study is the fact that patients are medicated. It is highly desirable to evaluate the performance of the proposed method on diagnosed but not yet medicated schizophrenia patients. It has been shown that antipsychotic medications have a normalizing effect on the functionality of the schizophrenia patients' brain (Davis et al., 2005). Moreover, prior fMRI and EEG studies on not medicated schizophrenia patients have reported altered FC (Omori et al., 1995; Meyer-Lindenberg et al., 2005).

It has been shown that the main targets of antipsychotic treatments in schizophrenia patients are cortical and subcortical motor networks (Wenz et al., 1994; Muller et al., 2003; Rogowska et al., 2004; Abbott et al., 2011). Recently the effect of antipsychotic treatment on resting-state FNC was studied (Lui et al., 2010) and it was shown that after treatment patients showed three connectivity changes compared to healthy controls. From these three changes only one (FNC between the temporal and parietal network) was present in this study. To further reduce the effect of medication on classification results, we repeated the classification with all described methods on reduced set of features where the motor network related features along with temporal-parietal FNC feature were excluded.

## Results

**Figure 2:**
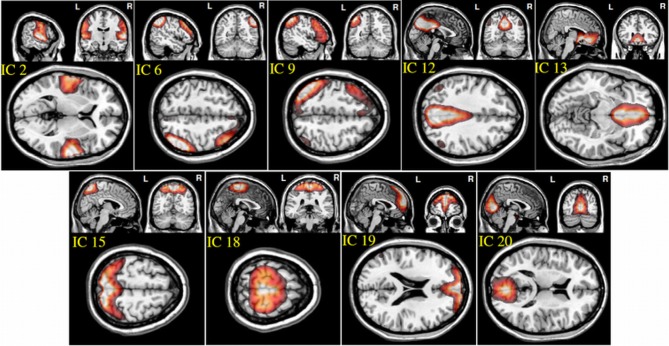
**Spatial maps of the nine selected IC components**.

From the 20 ICA components, 9 components were selected as non-artifactual, relevant networks. Since we selected nine IC components and we were interested in connectivity between each pair of networks, we ended up with 36 FNC features for each subject $\left(\begin{array}{c} 9\\ 2 \end{array}\right)$. Figure 2 illustrates the SMs of the selected IC components. These networks are: auditory network (IC #2), frontal-parietal networks (IC #6 and 9), default-mode networks (IC #12, 13, and 19), visual networks (IC #15 and 20) and motor network (IC # 18). Detailed information of each spatial map such as regions of activation, Brodmann area, volume and peak activation *t*-value and coordinates are provided in Table 1.

**Table 1:** **Brain regions, corresponding Brodmann areas, volumes, maximum *t*-values and spatial coordinates of each component in talairach space**.

	**BA**	**Vol**.	***T*_max_**	***X-Y-Z* coordinates**
**ATTENTIONAL NETWORKS**				
**IC 6**				
R middle frontal gyrus	8	35.2	22.3	(45, 40, −10)
R inferior parietal lobule	40	16.6	27.3	(45, −62, 39)
R inferior frontal gyrus	44, 45	28.1	19.5	(48, 40, −12)
R superior frontal gyrus	6, 8, 9	23.1	16.4	(39, 17, 49)
**IC 9**				
L middle frontal gyrus	8	25.2	33.5	(−45, 31, 32)
L inferior parietal lobule	40	25.0	36.0	(−53, −41, 46)
L inferior frontal gyrus	44, 45	20.9	25.8	(−53, 27, 21)
L superior frontal gyrus	6, 8, 9	26.9	21.8	(−45, 37, 31)
**VISUAL NETWORKS**				
**IC 15**				
R/L superior parietal lobule	5, 7	7.0/6.1	36.4/31.2	(18, −64, 56)/(−15, −64, 53)
R/L precuneus	7	28.3/26.2	30.8/31.6	(9, −58, 61)/(−15, −67, 50)
R/L cuneus	7, 19	8.5/7.3	28.3/23.4	(21, −71, 31)/(30, −77, 31)
**IC 20**				
L/R cuneus	7, 19	21.2/24.8	32.8/40.2	(6, −67, 9)/(−6, −73, 6)
L/R lingual gyrus	18, 19	21.2/24.8	31.4/43.8	(9, −84, 2)/(−6, −76, 4)
**DEFAULT-MODE NETWORKS**				
**IC 12**				
R/L precuneus	7	27.6/27.1	45.1/33.7	(6, −51, 33)/(−3, −54, 33)
R/L cingulate gyrus	23, 24, 31	19.6/15.9	32.4/29.1	(9, −54, 28)/(−3, −45, 30)
**IC 13**				
R/L anterior cingulate cortex	32	10.2/11.6	29.3/34.5	(6, 26, −6)/(−6, 26, −4)
R/ L medial frontal gyrus	9, 10	13.6/12.9	22.3/23.9	(12, 40, −10)/(−9, 38, −7)
**IC 19**				
R/L superior frontal gyrus	6, 8, 9	27.0/27.6	33.5/32.2	(9, 62, 16)/(−6, 59, 22)
R/L medial frontal gyrus	8, 9, 10	14.7/16.6	31.0/25.6	(3, 50, 17)/(−3, 44, 14)
**MOTOR NETWORK**				
**IC 18**				
R/L precentral gyrus	4, 6	11.9/11.8	23.0/32.2	(12, −20, 67)/(−30, −20, 56)
R/L medial frontal gyrus	6, 32	10.6/9.6	29.2/29.2	(6, −8, 64)/(−9, −23, 59)
R/L postcentral gyrus	1, 2, 3	11.8/12.2	24.3/32.2	(15, −38, 60)/(−15, −38, 60)
**AUDITORY NETWORK**				
**IC 2**				
R/L superior temporal gyrus	22	23.6/19.1	27.8/23.8	(50, −14, 6)/(−48, −17, 9)
R/L postcentral gyrus	1, 2, 3	16.1/16.4	24.1/20.3	(59, −20, 15)/(−50, −14, 17)
R/L insula	13, 47	12.6/13.1	23.6/22.7	(42, −8, 6)/(−45, −17, 12)

**Figure 3:**
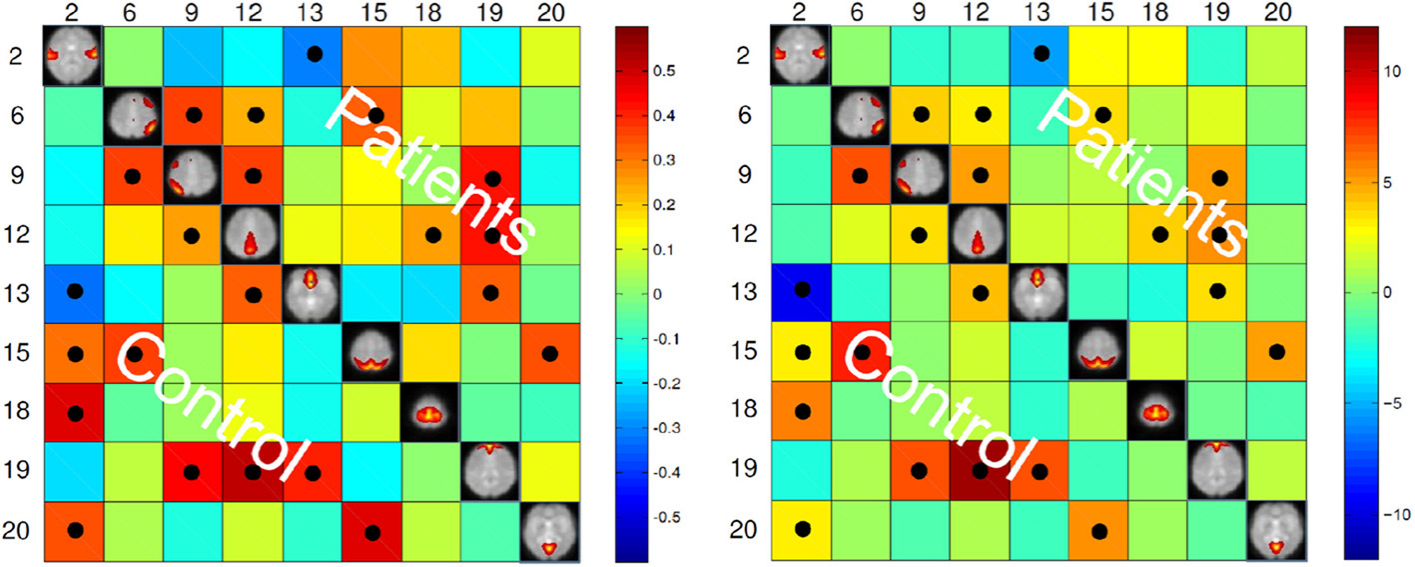
**Left:** Mean of correlation pairs for controls and patients. **Right:** *T*-value of each correlation pair resulted from student *t*-test with *p*-value threshold of 0.05 corrected for FDR. Black circles indicate the pairs surviving the *t*-test.

**Figure 4:**
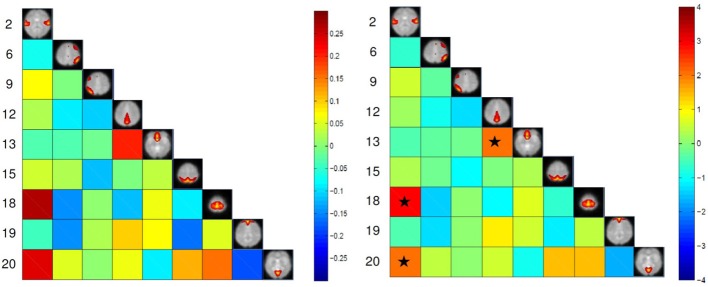
**Left:** Mean correlation difference between control subjects and patients (control-patient). **Right:** *T*-value resulting from two sample *t*-test with *p*-value threshold of 0.05 corrected for FDR. Stars show pairs that survived the paired *t*-test.

The maximum lagged correlation was computed for each of the subjects in each group. For each of the correlation pairs, student *t*-test was conducted with an FDR-corrected *p*-value threshold of 0.05 to identify significant correlations. Figure 3 shows the average correlation and the corresponding *t*-values. The black circles determine the correlation pairs that survived the *t*-test. It is seen that there are more significant correlation pairs (12) in the control group compared to patients group (10). Interestingly, the mean correlation between the auditory network (IC #2) with each of the visual networks (IC #15 and 20) and the motor network (IC #18) is significant for the healthy group but not for the patients. To determine which correlation pairs are significantly different between the two groups, two sample *t*-tests were conducted with a FDR corrected *p*-value threshold of 0.05. Also a mean correlation difference between the two groups (control-patients) was computed for each correlation pair. These results are shown in Figure 4. Starred pairs indicate those features surviving the paired *t*-test.

The classification results on the testing dataset for described classification methods (section Classification) are summarized in Table 2. For each method, overall classification accuracy, sensitivity, specificity, positive predicative value (PPN) and negative predictive value (NPV) are provided. Moreover, we reported the Wilson's binomial confidence interval (Wilson, 1927) for each classifier. For relevant methods, the choice of parameters selected during the training phase along with the topology of ANN s are also included in Table 2. As discussed in section Effect of Medication, to reduce the effect of medication on the classification results we repeated the analysis on the reduced set of features. Out of 36 features, 9 features that were more susceptible to medications were excluded from the feature set and the whole classification was repeated on the remaining 27 features. The excluded features are 8 motor related features (all FNC features involving IC18) along with a temporal-parietal feature (FNC between IC2 and IC15). The results are summarized in Table 3.

**Table 2:**
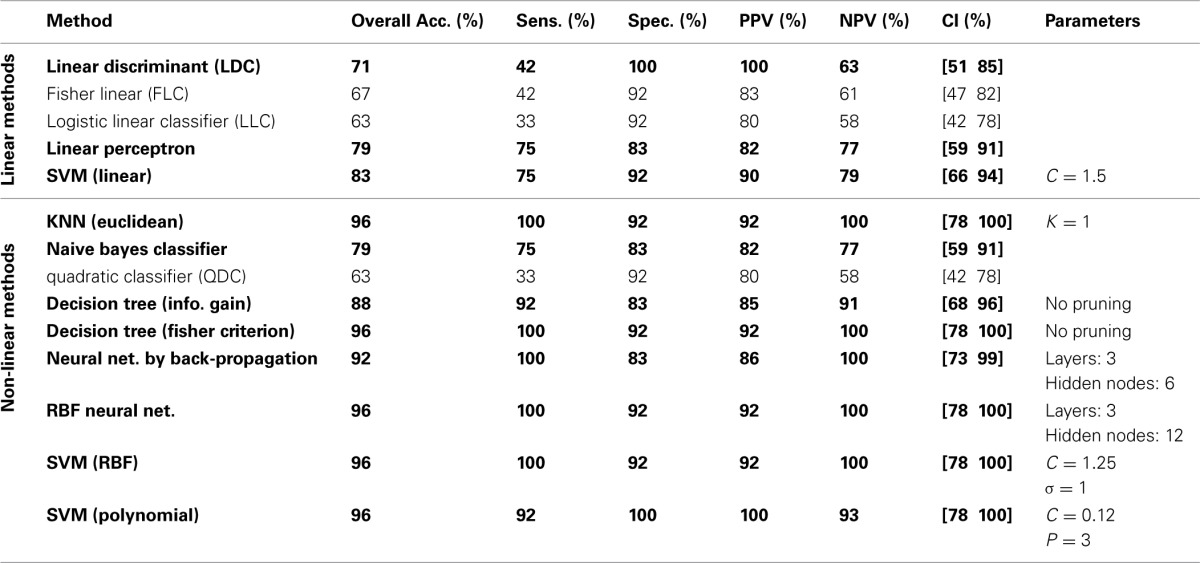
**Testing classification results using full set of features**.

Overall Acc, overall accuracy; Sens, sensitivity; Spec, Specificity; PPV, positive predictive value; NPV, negative predictive value; CI, Wilson's binomial confidence interval. Bold classifiers perform above the chance (lower bound of confidence interval is greater than 50%).

**Table 3:**
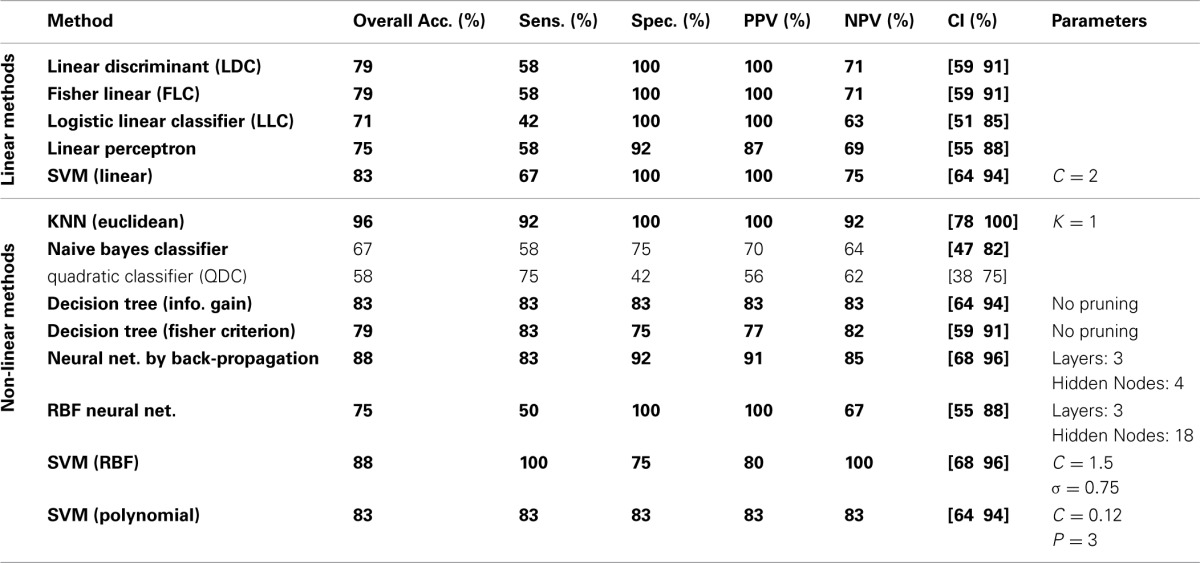
**Testing classification results using reduced set of features (27 features)**.

Overall Acc, overall accuracy; Sens, sensitivity; Spec, Specificity; PPV, positive predictive value; NPV, negative predictive value; CI, Wilson's binomial confidence interval. Bold classifiers perform above the chance (lower bound of confidence interval is greater than 50%).

**Figure 5:**
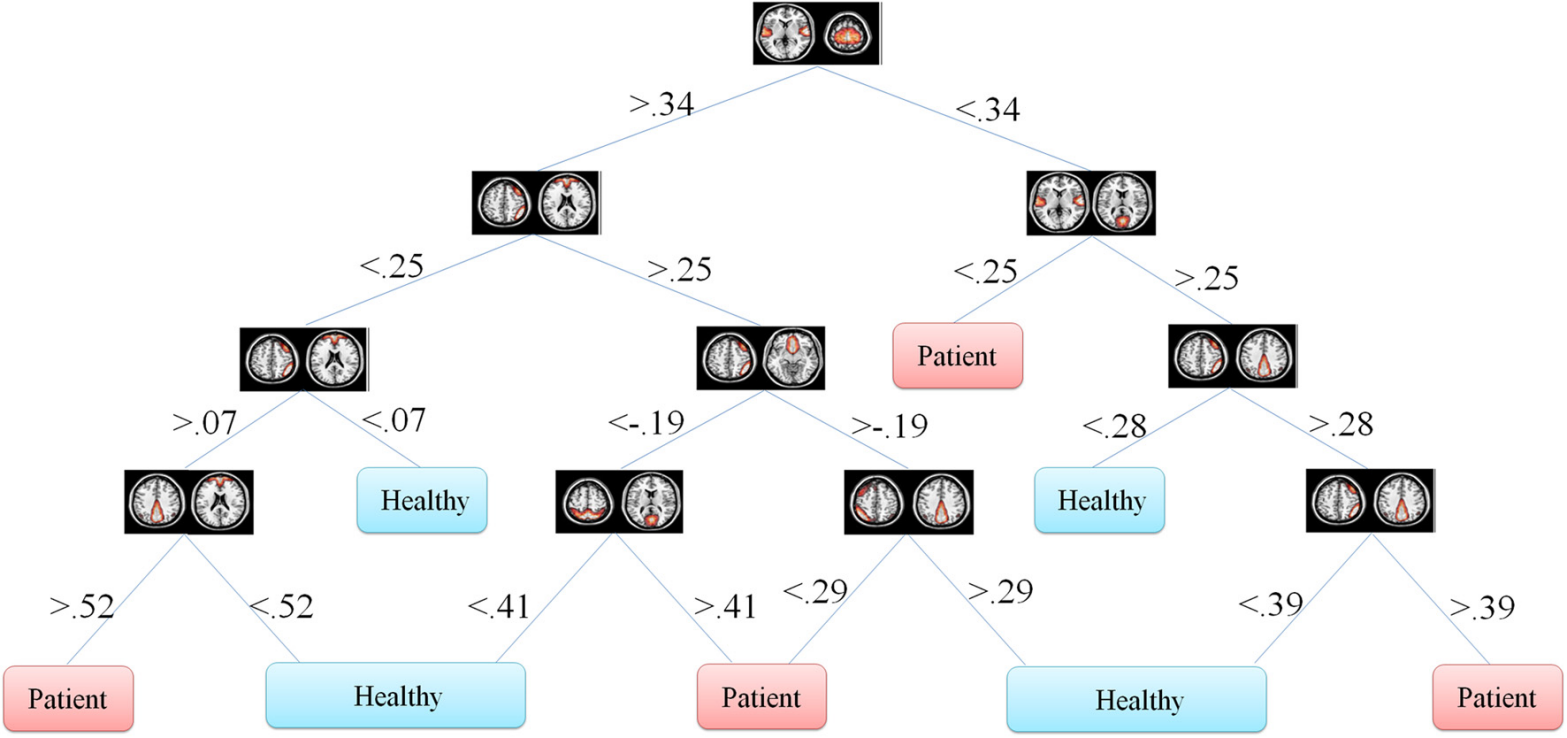
**Fisher's decision tree using full set of features**. This tree includes 8 features in 10 nodes.

**Figure 6:**
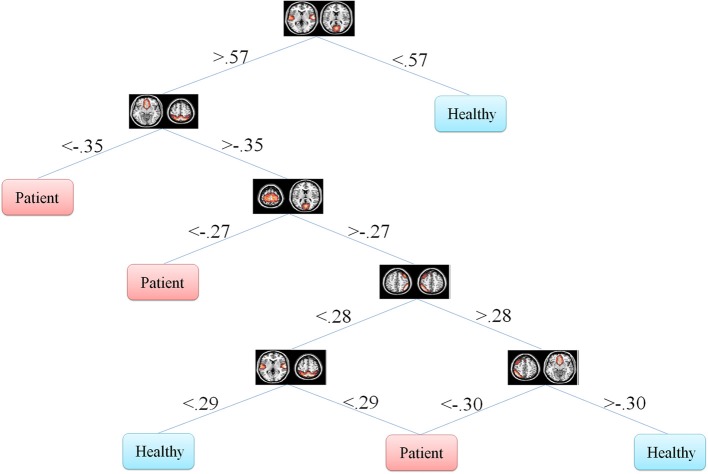
**Information gain decision tree using full set of features**. This tree includes six features in six nodes.

One of the main advantages of using DT is the graphical representation. One can represent decision alternatives and possible outcomes schematically. The visual approach is particularly helpful in comprehending sequential decisions and outcome dependencies. DT for both the Fisher's and information gain criteria are illustrated in Figures 5, 6, respectively.

## Discussion and conclusions

We investigated whether resting-state FC features are able to discriminate between schizophrenia patients and healthy control groups. Using group ICA, the training dataset was decomposed into independent spatial components and their corresponding TCs. Then, FNC was computed between each pair of functional networks on the back reconstructed data using the maximum lagged correlation method. Several linear and non-linear classifiers were trained using the training data and were evaluated using the testing data. One of the common pitfalls in classification of mental diseases is using cross-validation to measure the generalized error (Wood et al., 2007; Demirci et al., 2008b). Another pitfall is selection of parameter/model in a way that maximize the performance in the final classifier in the testing dataset (Demirci et al., 2008b). To avoid this, we used separate training and testing datasets. Separate ICAs were performed on training and testing datasets. Cross validation was used in the training phase just for parameter/model selection. ICA successfully extracted similar non-artifactual networks from both training and testing datasets. This not surprising since it has been shown that there are several consistent functional networks across subjects in the resting state (Damoiseaux et al., 2006; Smith et al., 2009; Allen et al., 2011).

The high accuracy of different classifiers in this study consolidates the disconnection hypothesis in schizophrenia patients (Friston and Frith, 1995; Frith et al., 1995; Josin and Liddle, 2001; Bokde et al., 2006; Mikula and Niebur, 2006; Salvador et al., 2010). Using FC methods, researchers have shown disrupted connectivity patterns in schizophrenia patients during rest and task in several brain regions (Meyer-Lindenberg et al., 2001; Boksman et al., 2005; Honey et al., 2005; Liang et al., 2006; Jafri et al., 2008). In our experiment, connectivity between two DMN nodes (IC #12 and 13) was found to be significantly lower in schizophrenia patients compared to healthy controls (Figure 4). This reduced within DMN connectivity is interesting and in line with recent findings (Camchong et al., 2011; Mingoia et al., 2012; Orliac et al., 2013). One explanation can be gray matter thinning and greater psychopathology in patients(Goghari et al., 2007; Jang et al., 2011). Some recent DTI studies have shown anatomical disconnection in several brain regions in temporal and frontal lobe in schizophrenia patients (Buchsbaum et al., 2006). Moreover some studies have associated anatomical damage and FC disconnection in patients by analyzing DTI and functional data together (Zhou et al., 2008). This anatomical-functional association may be the reason for successful automatic diagnosis studies using DTI (Caprihan et al., 2008; Ardekani et al., 2011) and fMRI studies (Georgopoulos et al., 2007; Calhoun et al., 2008b; Demirci et al., 2008a; Michael et al., 2008; Arribas et al., 2010; Shen et al., 2010). While anatomical studies using either DTI or structural MRI are popular in classification of schizophrenia patients, functional studies are limited mostly to task-based studies. Resting-state studies in case of classification of schizophrenia are rare and have been just recently started (Shen et al., 2010; Venkataraman et al., 2012). Most of the connectivity fMRI studies (resting-state or task-based) have used FC features which means that the features are temporal statistical dependencies among brain regions. Using FC methods have some limitation such as the choice of seed-voxel in each region (that may be different for patients and controls) and very high number of extracted features. Shen et al., extracted average time-courses from 116 brain regions which means 6670 features for each subject. High number of features requires additional step such as feature selection and reduction to avoid curse of dimensionality. Moreover, most of the features in that fashion are not discriminative. Using FNC on the other hand, doesn't require seed-voxel selection. Moreover, the number of extracted features is much less than FC methods (36 features in our experiment based on 9 functional networks). Based on our experiments, it can be inferred that FNC methodology is a concise abstraction of the connectivity pattern in the brain that can successfully capture the differences between schizophrenia patients from healthy controls.

We have reported detailed classification results (sensitivity, specificity, positive predictive value and negative predictive value) as well as Wilson's binomial confidence interval for each classification method. The classification results in Table 2 show that non-linear methods outperform linear methods, which was expected. Among the linear methods, LDC, Perceptron and linear SVM performed above the chance (lower bound of Wilson's binomial confidence interval is greater than 50%). All linear methods show high specificity than sensitivity. Except for quadratic classifier, all non-linear methods, performed above the chance. In overall, discriminative approaches outperformed generative methods. As a general rule in this study, the less assumptions about the data, the better the performance. Simple classifiers such as KNN and decision tree performed very well on this specific machine learning problem. Also, non-linear SVM showed significant performance with only one misclassified sample. Despite of oversimplified assumptions and little training data available in this study, the performance of naïve Bayes is marginally above the chance (79.17% overall performance). A poor classification was achieved using the quadratic classifier. It can be hypothesized that whether the assumptions of this classifier that two classes are normally distributed with different mean and covariance matrices are not valid or small amount of data is not sufficient to accurately estimate the mean and covariance matrix of each classifier. It should be noted that conclusions regarding the performance of different classifiers are limited to this specific problem using one dataset. Performance of each machine learning algorithm depends on the dataset and comparison among different classifiers has been heavily investigated in the machine learning literature. Since our main goal is not comparing classifiers, we didn't conduct statistical tests to compare their performances and just reported Wilson's binomial score interval for each classifier.

Table 3 shows the result of classification on reduced set of features. Surprisingly, the overall error was reduced for all the linear methods except for linear perceptron. The main reason for this phenomenon may be the curse of dimensionality (Pearlson, 2009) since we have only 32 samples for training and 36 features. Using the reduced feature set (27 features), most of the linear methods could estimate more accurate hyperplane. Linear SVM performs robustly and equally on both full and reduced set of features. Most non-linear classifiers still show above the chance performance with lower overall performance compared to the full feature set. KNN still classifies with high accuracy. Again, QDC performed very poorly. In overall, reduction of features didn't greatly affect the results and very high performances were still achievable. This suggests that medication didn't bias the classification.

DT don't transform the data from the original feature space. Moreover, they classify the data based on thresholds they put on each of the features. This makes it possible for the investigator to observe the decision tree and analyze it. One can see how features are distributed in different levels of the decision tree and what thresholds on which features discriminate the classes. This property is especially of interest in the medical diagnosis field since decision tree provides classification structure which includes thresholds on the symptoms. This discriminative information of each feature is very valuable in medical problems.

In our problem the symptoms are FNC features. One can observe that how each feature discriminate the two groups. This information may reflect FNC abnormalities in schizophrenia patients. First of all decision tree introduces the important features which are 8 and 6 in Figures 5, 6, respectively. Top node features are among the most important features which are among the feature identified by the two-sample *t*-test. Also, the decision tree can identify the type abnormality which is discriminative between the two groups. For example, it is seen from Figure 5 that subjects with temporal-motor FNC lower than 0.34 and temporal-visual higher than 0.25 are patients. Or from Figure 6 it is evident that all subjects with temporal-visual FNC lower than 0.57 are healthy controls. In other words, all patients have higher temporal-visual FNC (as do some of the healthy controls).

Figures 5, 6 illustrate Fisher's and information gain DT, respectively (full set of features). Fisher's decision tree includes eight features in nine nodes. Information gain decision tree includes six features in six nodes. It is interesting that using small subset of features, DT perform well in classification. Fisher's decision tree outperforms information gain tree when full set of feature are used but it is more complicated. However, both trees can be considered very simple. Using reduced set of features, information gain tree outperforms Fisher's tree.

Prior studies mentioned in the Introduction section reported accuracies ranging from 79 to 98%. Several limitations and considerations make it very hard to compare different approaches of automatic classification of mental disorders. For example, study size, MRI scanner parameters, nature of extracted features, type of classifier, medication and disease severity in the patient group varies among the different studies. In the absence of standard training and testing datasets, comparison of different approaches based only on the classification rate is ambiguous.

One of the issues in the current study was that the patients were slightly older than healthy controls. We looked at the misclassified subjects in each of the classification experiments and couldn't find any systematic age pattern. Note also, it has been shown that schizophrenia patients have stronger FNC (Jafri et al., 2008) whereas subjects that are older have reduced FNC (Allen et al., 2011). So, this potential confound would likely have a canceling effect making the diagnosis even harder. Regardless, based on the above observation we do not believe age is a factor in our classification results. To avoid any bias, we also repeated the classification when age was regressed out from the FNC features and exactly same performance was achieved.

In this study, we separated the data into training and testing dataset. One may wonder how our method works in a clinical situation when we have only one new subject. We assume that we have trained our model using enough training data. In this situation here are two options: (1) we can use the group ICA components of the training data as regressors and calculate the subject specific time-courses. (2) For a more accurate estimation another ICA can be done on an extended dataset containing training data and the new subject data. Note that we won't use the information of this new ICA analysis for training the classifiers/models but just to extract IC networks/time-series for the new test subject. This approach is more accurate but slower especially in the case of big training data. Since the main goal of this paper is to study the feasibility of using FNC features, we didn't investigate methods.

In this study we showed that the resting state FNC features can be successfully exploited in order to automatically discriminate schizophrenia patients. To the best of our knowledge this the first study using resting-state FNC features to classify schizophrenia patients. Acquiring scans from schizophrenia patients is more feasible in the resting state due to the short acquisition time and avoidance of cognitive task-related impairment confounds. Moreover, the data is less prone to multi-site variability (Pearlson and Calhoun, 2009). It was demonstrated that just 5 min resting state data can be used to classify patients reliably and accurately using FNC features and simple classifiers such as KNN. Moreover, performance of several linear and non-linear methods were evaluated and compared.

### Conflict of interest statement

The authors declare that the research was conducted in the absence of any commercial or financial relationships that could be construed as a potential conflict of interest.
